# Can anticipated regret promote rationality? The influence of anticipated regret on risk aversion and choice satisfaction

**DOI:** 10.3389/fpsyg.2025.1667136

**Published:** 2025-09-11

**Authors:** Jing Liu, Haiyan Liu

**Affiliations:** ^1^School of Psychology, Hainan Normal University, Haikou, China; ^2^Adolescent Psychological Development and Education Center of Hainan, Haikou, China

**Keywords:** anticipated regret, risk aversion, choice satisfaction, risk preference, time pressure, Balloon Analog Risk Task

## Abstract

**Objective:**

This research examines the impact of anticipated regret on decision-making under risk, focusing specifically on its role in promoting risk aversion and enhancing choice satisfaction.

**Methods:**

Three studies were conducted to systematically investigate the effects of anticipated regret. In Study 1, the Balloon Analog Risk Task (BART) was used to manipulate the presence or absence of anticipated regret. Study 2 introduced individual differences in risk preference to examine how this trait interacts with anticipated regret in shaping risk-taking behavior. Study 3 added a time pressure condition to explore how anticipated regret functions under varying decision-making constraints.

**Results:**

Study 1 showed that anticipated regret significantly increased risk-averse behavior and improved choice satisfaction. In Study 2, both anticipated regret and individual risk preference influenced risk-taking, but only anticipated regret had a consistent positive effect on satisfaction. No significant interaction was found between the two variables. Study 3 revealed that participants were generally more risk-averse under time pressure, and the effect of anticipated regret on risk avoidance was attenuated in high-pressure conditions. However, its positive influence on satisfaction remained stable across conditions.

**Conclusion:**

Anticipated regret consistently influenced both risk-taking behavior and choice satisfaction across different individual dispositions and situational conditions, highlighting the stable and significant role of prospective emotions in decision-making under risk.

## 1 Introduction

In daily life, individuals frequently face decisions involving risk—from financial and career choices to health and everyday consumption—often made under uncertainty and eliciting strong emotional responses. Regret is typically experienced after a decision as a negative emotion, but it can also be anticipated, influencing option evaluation beforehand (Zeelenberg and Pieters, 2007; Brewer et al., 2016). Research indicates that anticipated regret promotes cautious, risk-averse behavior (Richard et al., 1996) and affects post-decision satisfaction (Inbar et al., 2011; Li et al., 2022). Moreover, recent evidence shows that emotional cues, framing, and motivational contexts shape how anticipated regret operates in decision-making. Specifically, anticipated negative emotions influence choices in moral dilemmas (Pletti et al., 2016), framing and emotional information shape decision outcomes (Palmiotti et al., 2020), motivational contexts affect adaptive cognitive control in children (Toffoli et al., 2025), and impulsivity and reward sensitivity impact moral decisions under time pressure (Del Popolo Cristaldi et al., 2024). However, few studies have simultaneously examined its effects on risk-taking behavior and choice satisfaction or systematically explored how stable traits (e.g., risk preference) and situational constraints (e.g., time pressure) shape decision processes. The present study aims to address these gaps and provide an integrated framework for understanding how emotional, dispositional, and contextual factors influence decisions and subsequent evaluations.

Building on these empirical findings, anticipated regret can be conceptualized as a forward-looking, regulatory emotional forecast. According to regret theory, decisions are influenced not only by expected outcomes but also by counterfactual comparisons with unchosen alternatives (Bell, 1982; Loomes and Sugden, 1982). Decision affect theory posits that individuals automatically anticipate emotions such as regret and disappointment when evaluating possible outcomes, which systematically shape their choices (Mellers, 2000). In risk-related contexts, anticipated regret typically increases risk aversion, as individuals trade potential gains for emotional security (Zeelenberg and Pieters, 2007; Beattie et al., 1994). Neuroimaging studies support these findings, showing functional interactions between prefrontal and limbic regions during anticipated regret (Liu et al., 2023).

Although risk aversion is often viewed as relatively stable, it is also shaped by personality traits, emotional states, and social contexts (Kahneman and Tversky, 2013; Feldman Hall and Shenhav, 2019). Choice satisfaction, the subjective evaluation of one’s decision outcomes, is another key indicator of decision quality (Sweeny and Vohs, 2012). Anticipated regret not only affects the decisions people make but also post-decision satisfaction (Zeelenberg et al., 2000; Camille et al., 2004; Pletti et al., 2016). For instance, avoiding risky options may increase satisfaction by reinforcing a sense of responsibility or moral justification, whereas excessive risk aversion could limit opportunities for better outcomes, reducing satisfaction (Connolly and Zeelenberg, 2002; Brewer et al., 2016).

To provide a coherent theoretical framework, the present study focuses on three key variables: anticipated regret, individual risk preference, and time pressure. Anticipated regret is treated as the primary emotional factor, expected to influence both risk-averse behavior and post-decision satisfaction, consistent with regret theory and decision affect theory (Zeelenberg and Pieters, 2007; Mellers, 2000). Individual risk preference is included as a stable dispositional variable that may shape the impact of anticipated regret, such that individuals with lower risk tolerance are likely to exhibit stronger effects on both risk-taking and satisfaction outcomes (Weber et al., 2002; Blais and Weber, 2006). Time pressure is incorporated as a situational factor that may constrain counterfactual thinking and deliberation, potentially weakening or amplifying the effect of anticipated regret depending on task conditions (Suri and Gross, 2012; Wu et al., 2022). Importantly, these three variables are conceptually linked: anticipated regret acts as the proximal emotional mechanism driving behavior and satisfaction, while individual risk preference and time pressure determine the conditions under which this emotion manifests in observable decision patterns. By explicitly modeling these interactions, the study provides a theoretically grounded account of how emotional, dispositional, and contextual factors influence risk-taking behavior and post-decision evaluations.

### 1.1 Anticipated regret and risk aversion

Anticipated regret is a key factor influencing risk-averse behavior. In the Balloon Analog Risk Task (BART), individuals anticipating negative consequences of a balloon burst—particularly regret—tend to stop inflating earlier, resulting in fewer pumps (Lejuez et al., 2002; Zeelenberg et al., 1996). Emotional recall and reflective prompts further amplify risk avoidance (Lee and Aaker, 2004; Cavlovic et al., 2025). Neuroimaging findings indicate that anticipated regret engages cognitive control circuits, providing physiological evidence for its regulatory role (Liu et al., 2023). Based on this literature, we hypothesize:

*H1*: Participants in the anticipated regret condition will exhibit greater risk aversion, as reflected in fewer balloon pumps, compared to those in a control condition.

### 1.2 Anticipated regret and choice satisfaction

Anticipated regret also influences post-decision satisfaction. While regret is typically negative, anticipating it can enhance acceptance of decisions (Inbar et al., 2011). Emotional engagement in decision-making may promote post-choice rationalization, increasing satisfaction even when outcomes are suboptimal (Tsiros and Mittal, 2000; Li et al., 2022; Pletti et al., 2016). Recent studies further show that contextual framing, impulsivity, and motivational factors shape how anticipated regret affects satisfaction (Palmiotti et al., 2020; Toffoli et al., 2025; Del Popolo Cristaldi et al., 2024). Based on these findings:

*H2*: Participants anticipating regret will report higher choice satisfaction compared to participants in the control condition.

### 1.3 Individual risk preference and decision-making behavior

Risk preference, a stable trait reflecting tolerance for uncertainty, shapes decision behavior (Weber et al., 2002; Figner and Weber, 2011). High-risk individuals pursue uncertain options, while low-risk individuals prefer conservative strategies. Prior research indicates that risk preference may moderate the influence of anticipated regret, though results are mixed (Blais and Weber, 2006; Wu et al., 2022; Jin et al., 2025). Therefore:

*H3*: The effect of anticipated regret on risk aversion and choice satisfaction may vary depending on individual risk preference, such that low-risk participants are more strongly influenced by anticipated regret than high-risk participants.

### 1.4 Boundary conditions of anticipated regret: the role of time pressure

Situational factors, such as time constraints, can limit counterfactual thinking and deliberation, potentially attenuating the regulatory role of anticipated regret (Zur and Breznitz, 1981; Suri and Gross, 2012; Toffoli et al., 2025; Del Popolo Cristaldi et al., 2024). Conversely, when tasks provide feedback, time pressure may amplify conservative behavior (Wu et al., 2022). Thus:

*H4*: The influence of anticipated regret on risk aversion and choice satisfaction may vary depending on time pressure, with stronger effects observed under low time pressure conditions.

### 1.5 Mechanisms of anticipated regret

Anticipated regret occurs before a decision, prompting careful evaluation of alternatives and avoidance of unfavorable outcomes (Simonson, 1992; Zeelenberg and Pieters, 2007). Its regulatory power is shaped by induction methods, cognitive constraints, and task structure (Richard et al., 1996; Shiv and Fedorikhin, 1999; Inbar et al., 2011; Krönke et al., 2020).

While prior studies have demonstrated that anticipated regret influences both risk-taking and satisfaction, important gaps remain. Most research has examined these outcomes separately, leaving unclear how regret simultaneously shapes decision behavior and post-decision evaluations within the same paradigm. Moreover, although the Balloon Analog Risk Task (BART) offers ecologically valid risk–reward tradeoffs, few studies have directly linked anticipated regret to behavioral outcomes in this task. Finally, the moderating roles of stable traits (e.g., individual risk preference) and situational factors (e.g., time pressure) have been suggested but not systematically tested together. The present study addresses these issues by investigating the joint effects of anticipated regret on risk aversion and choice satisfaction in the BART, while testing how individual risk preference and time pressure shape these processes. In doing so, this work extends existing research by moving beyond documenting main effects to clarifying the boundary conditions under which regret regulates decision-making.

Overall, anticipated regret functions as a forward-looking, adaptive mechanism that regulates both risk-taking and post-decision evaluations.

## 2 Study 1: Materials and methods

### 2.1 Participants

*A priori* power analysis was conducted using G*Power 3.1.9.7. Based on a medium effect size (Cohen’s *d* = 0.5), with *α* = 0.05 and statistical power set at 0.80, the required sample size was estimated to be 128 participants. Across all studies, participants reported good physical and mental health and had normal or corrected-to-normal vision. Data quality was ensured by excluding participants with incomplete data or abnormal responses (i.e., extremely rapid task completion or repetitive, mechanical actions in the BART). A total of 135 undergraduate students participated in the study. Two participants were excluded due to incomplete data or abnormal responses, yielding a final sample of 133 (71 females; *M* = 19.34 years, *SD* = 1.62). Participants were randomly assigned to either the anticipated regret condition (*n* = 66) or the control condition (*n* = 67). This study was approved by the Ethics Committee of the School of Psychology, Hainan Normal University.

### 2.2 Design

The experiment employed a between-subjects design with one independent variable: anticipated regret (present vs. absent). The dependent variables were risk-averse behavior and choice satisfaction. Risk-averse behavior was operationalized as the average number of balloon pumps on non-exploded trials in the BART task—fewer pumps indicated a higher level of risk aversion. Choice satisfaction was measured through a post-task self-report using a 7-point Likert scale to assess participants’ subjective evaluation of their decision outcomes.

### 2.3 Procedure

**Figure 1:**
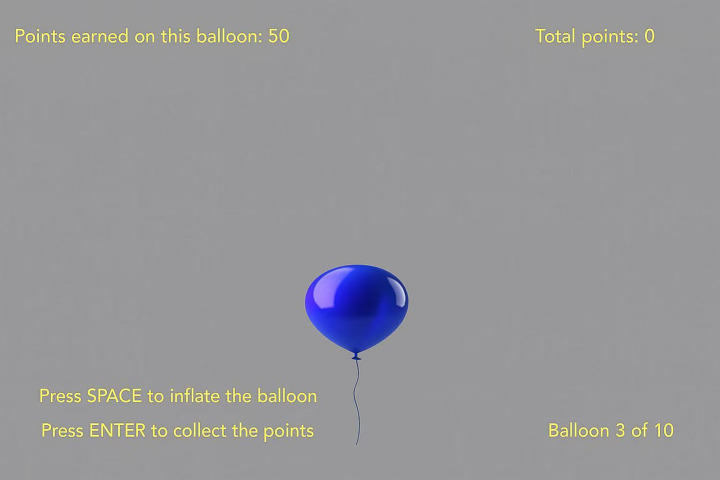
Balloon Analog Risk Task (BART) interface.

The experimental paradigm was adapted from the Balloon Analog Risk Task (BART) (Figure 1), a widely used behavioral measure of risk-taking (Lejuez et al., 2002). In this computerized task, participants made repeated decisions about whether to inflate a virtual balloon. Each pump increased the potential reward by 10 points, but the balloon could explode at a random threshold, resulting in the loss of all points for that trial. If participants chose to stop inflating before an explosion, the accumulated points were added to their total score. To manipulate anticipated regret, an emotion-eliciting induction procedure adapted from Zeelenberg et al. (1998) was used. Participants in the anticipated regret condition completed two priming tasks: they first recalled and described a past situation in which they experienced strong regret due to a poor decision, and then imagined how regretful they would feel if they made a risky choice and lost all rewards during the task. Participants in the control condition completed a structurally similar but emotionally neutral task by recalling a recent routine activity (e.g., taking public transportation or walking). This ensured that both groups engaged in similar task-related cognitive activities, while only the regret group received emotional priming.

The experiment was administered online via personal computers. Participants were recruited through social media platforms and accessed the study through a secure link. After reading an overview of the study and providing informed consent, they were randomly assigned to either the regret or control condition. Following the priming phase, all participants completed three practice trials of the BART to become familiar with the task mechanics. The formal session included 10 experimental trials, during which participants decided whether to continue inflating a balloon to increase rewards or stop and secure their current earnings. Each action was followed by immediate visual and numerical feedback indicating success or explosion. Upon completing the task, participants rated their choice satisfaction on a 7-point Likert scale. The entire procedure was implemented using PsychoPy version 2021.2.3, ensuring consistent presentation and data collection.

### 2.4 Results and analysis

All statistical analyses were performed using SPSS 27.0, Independent samples *t*-tests were conducted to examine differences between the anticipated regret and control groups on two key outcome variables: risk-averse behavior and choice satisfaction.

#### 2.4.1 Risk aversion analysis

**Figure 2:**
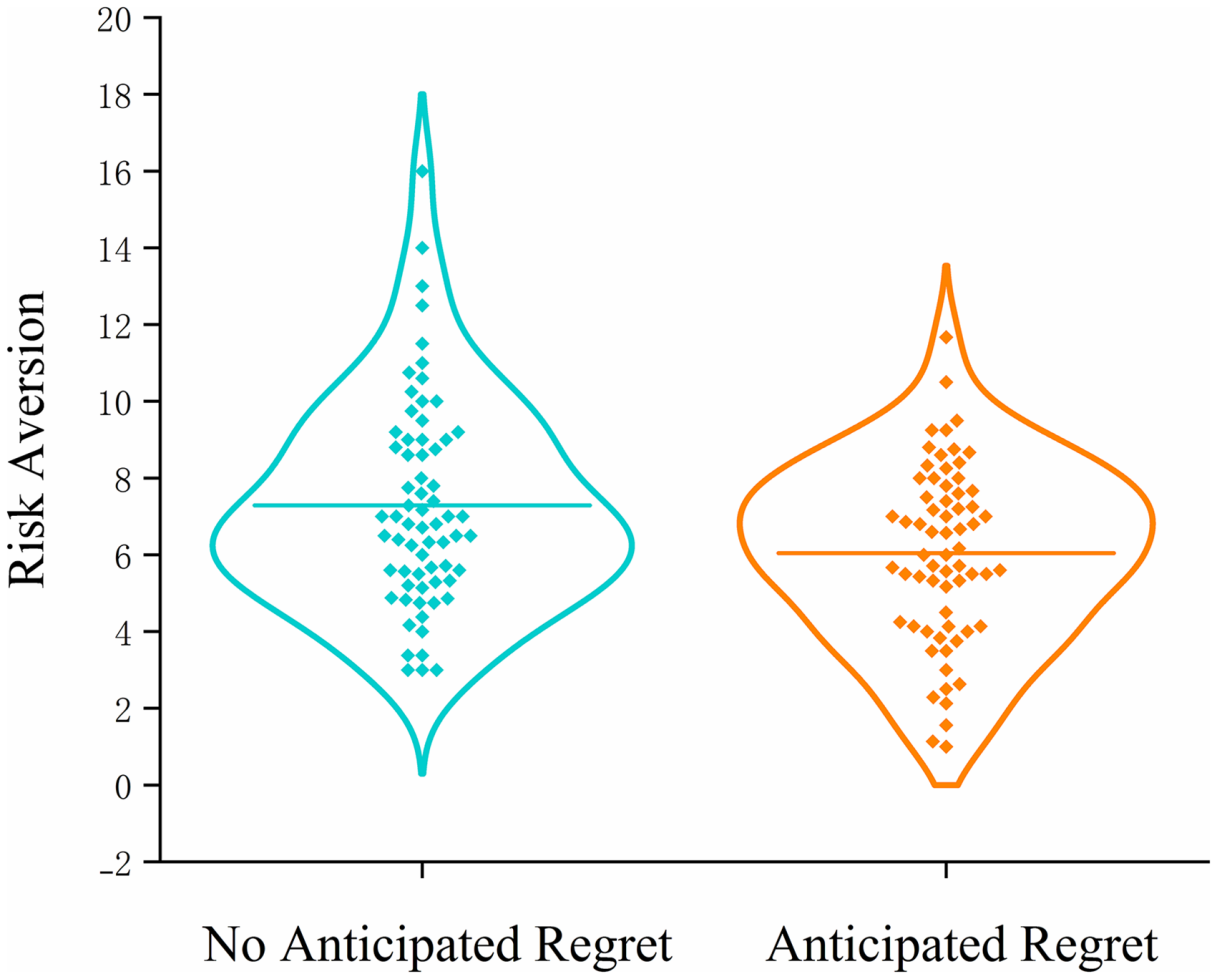
Violin plots of risk-averse behavior in Study 1 under anticipated regret (present vs. absent) conditions.

Participants in the anticipated regret condition (*M* = 6.05, SD = 2.31) exhibited significantly fewer pumps than those in the control group (*M* = 7.29, SD = 2.70), *t*_131_ = 2.84, *p* = 0.005, Cohen’s *d* = 0.49, 95%CI (0.15, 0.84). This finding suggests that the induction of anticipated regret promoted greater risk aversion, as evidenced by more cautious balloon inflation behavior (Figure 2).

#### 2.4.2 Choice satisfaction analysis

**Figure 3:**
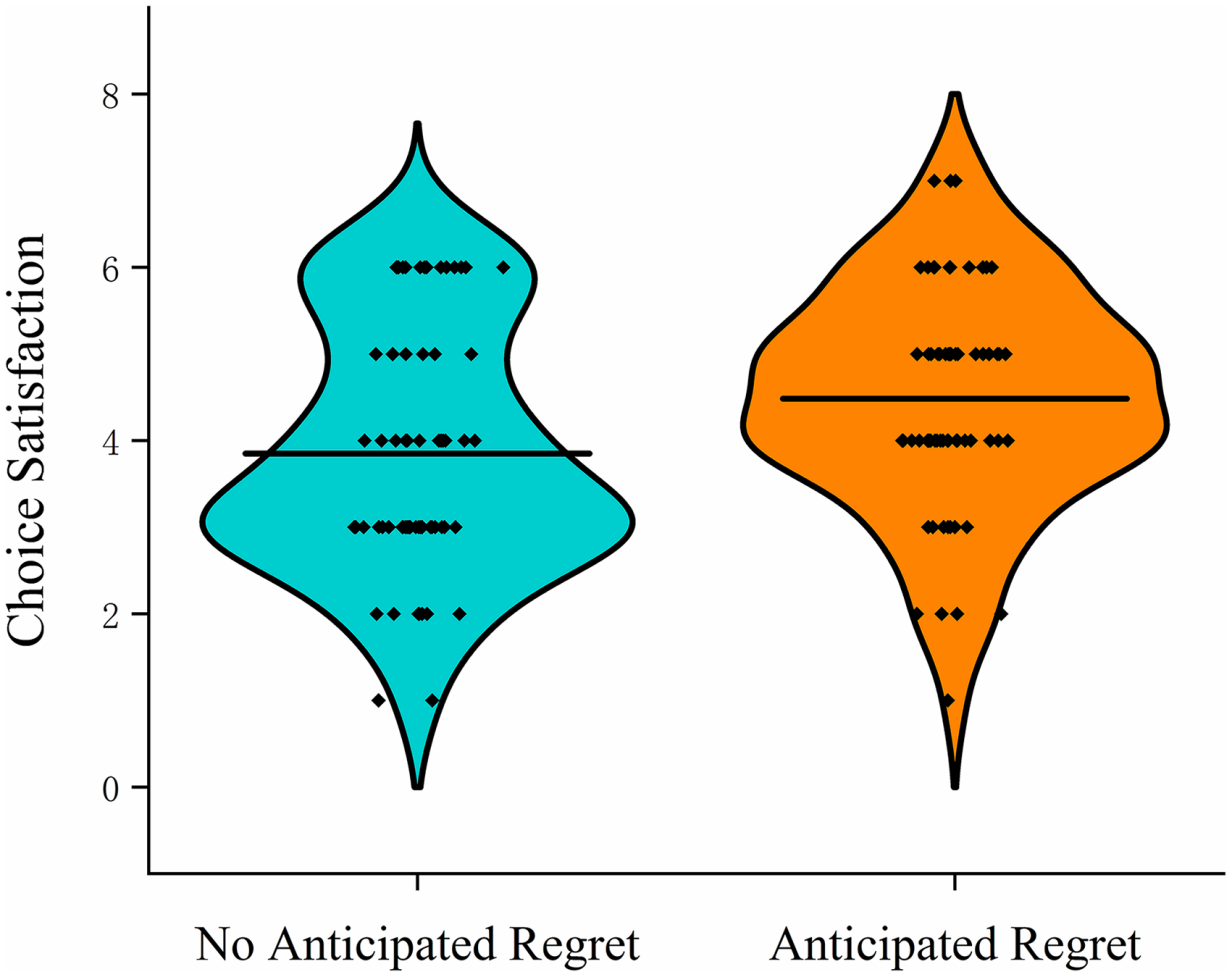
Violin plots of choice satisfaction in Study 1 under anticipated regret (present vs. absent) conditions.

Participants in the anticipated regret condition (*M* = 4.48, SD = 1.28) reported significantly higher satisfaction with their decision-making process than those in the control group (*M* = 3.85, SD = 1.42), *t*_131_ = −2.71, *p* = 0.008, Cohen’s *d* = −0.47, 95%CI (−0.81,−0.12). These findings suggest that a moderate level of prospective emotion, such as anticipated regret, may facilitate more favorable evaluations of one’s choices (Figure 3).

### 2.5 Summary

The results of Study 1 demonstrated that anticipated regret leads to increased risk-averse behavior and greater satisfaction with decision outcomes. Although regret is commonly seen as a negative emotion, its anticipation before making decisions appears to encourage more cautious choices and more positive evaluations of those choices afterward.

These findings align with earlier studies that emphasize the adaptive function of anticipated emotions in guiding behavior (Richard et al., 1996; Zeelenberg et al., 2000). They also support the affective forecasting theory, which suggests that people mentally simulate the emotional impact of possible future outcomes and adjust their behavior to avoid negative feelings (Zeelenberg and Pieters, 2007).

## 3 Study 2: Materials and methods

### 3.1 Participants

The general methodology was identical to Study 1, except for the following differences. *A priori* power analysis using G*Power 3.1.9.7 indicated that a minimum of 128 participants would be required to detect a medium effect size (*f* = 0.25) with *α* = 0.05 and power = 0.80 in a 2 × 2 factorial design. A total of 138 undergraduate students took part in the study. Two participants were excluded due to incomplete data or abnormal responses, resulting in a final sample of 136 (72 females; *M* = 19.35 years, *SD* = 1.61). Participants were randomly assigned to one of four experimental conditions: high risk preference with anticipated regret (*n* = 33), low risk preference with anticipated regret (*n* = 33), high risk preference without anticipated regret (*n* = 35), and low risk preference without anticipated regret (*n* = 35).

### 3.2 Design

This study used a 2 (anticipated regret: present vs. absent) × 2 (risk preference: high vs. low) between-subjects factorial design. The primary difference from Study 1 was the inclusion of participants’ dispositional risk preference as a second independent variable, allowing us to examine whether risk preference altered the effect of anticipated regret on risk-averse behavior and choice satisfaction.

### 3.3 Procedure

To further examine the influence of anticipated regret on decision-making, Study 2 was designed to explore whether individuals’ risk tendencies shape their behavioral and subjective responses to anticipated regret. Specifically, we hypothesized that participants with higher or lower risk preferences might display different patterns of risk-taking and choice satisfaction when anticipating potential regret. To investigate this, risk preference was incorporated as an experimental factor, allowing the study to examine how anticipated regret interacts with dispositional risk tendencies in influencing both risk-averse behavior and subjective evaluations. Based on this rationale, the experimental conditions and procedure were designed to systematically test these predictions.

The main difference from Study 1 was the assessment of individual risk preference using the Risk Propensity Scale (RPS; full items listed in Appendix; Meertens and Lion, 2008). This seven-item self-report instrument evaluates general tendencies toward risk-seeking behavior on a Likert scale, with higher scores indicating stronger risk-seeking propensities. The scale demonstrated acceptable internal consistency in the current sample (Cronbach’s *α* = 0.78). To facilitate group comparisons, participants were classified into high and low risk preference groups using a median split of RPS scores (median = 26), a common approach in prior research when sample sizes are moderate and the focus is on contrasting extreme tendencies (Iacobucci et al., 2015a, 2015b). This method is straightforward and intuitive, allowing for clear comparisons between participants with relatively high versus low risk preferences, and it is robust to extreme values, helping to reduce the influence of outliers while maintaining roughly balanced group sizes. Otherwise, the experimental paradigm, emotional induction, BART procedure, and attention checks were identical to Study 1.

### 3.4 Results and analysis

Data were analyzed using two-way analyses of variance (ANOVAs) to examine the main and interaction effects of anticipated regret (present vs. absent) and risk preference (high vs. low) on the same outcome variables.

#### 3.4.1 Risk aversion analysis

**Figure 4:**
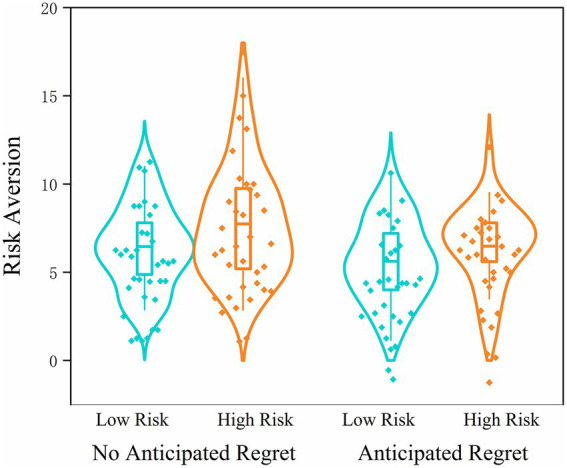
Violin plots of risk-averse behavior in Study 2 under different anticipated regret (present vs. absent) and risk preference (high vs. low) conditions.

The ANOVA results revealed a significant main effect of anticipated regret, *F*(1, 132) = 5.87, *p* = 0.017, η_p_^2^ = 0.043. Participants in the anticipated regret condition (high risk preference: *M* = 6.48, SD = 2.26; low risk preference: *M* = 5.62, SD = 2.30) made significantly fewer pumps than those in the no-regret condition (high risk preference: *M* = 7.74, SD = 3.12; low risk preference: *M* = 6.46, SD = 2.29), indicating greater risk aversion when regret was anticipated. This finding is consistent with the results observed in Study 1. A significant main effect of risk preference also emerged, *F*(1, 132) = 6.12, *p* = 0.015, η_p_^2^ = 0.044. Participants with low risk preference showed more conservative pumping behavior than those with high risk preference, confirming the influence of dispositional risk tendency on behavioral choices. However, the interaction between anticipated regret and risk preference was not statistically significant, *F*(1, 132) = 0.24, *p* = 0.629, η_p_^2^ = 0.002. This suggests that the effect of anticipated regret on risk aversion was consistent regardless of participants’ individual risk preference (Figure 4).

#### 3.4.2 Choice satisfaction analysis

**Figure 5:**
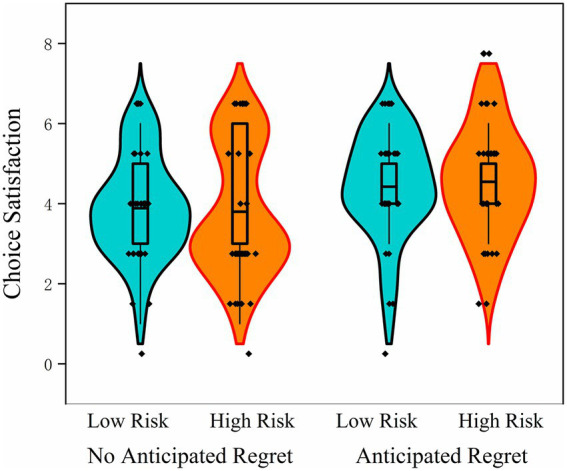
Violin plots of choice satisfaction in Study 2 under different anticipated regret (present vs. absent) and risk preference (high vs. low) conditions.

The ANOVA results revealed a significant main effect of anticipated regret, *F*(1, 132) = 7.72, *p* = 0.006, η_p_^2^ = 0.055. Participants in the anticipated regret condition (high risk preference: *M* = 4.55, SD = 1.33; low risk preference: *M* = 4.42, SD = 1.25) reported significantly higher choice satisfaction than those in the no-regret condition (high risk preference: *M* = 3.80, SD = 1.55; low risk preference: *M* = 3.89, SD = 1.23). This suggests that inducing anticipated regret prior to decision-making can effectively enhance individuals’ satisfaction with their choices, consistent with the pattern observed in Study 1. In contrast, the main effect of risk preference was not significant. Participants with high risk preference did not differ significantly in choice satisfaction from those with low risk preference, *F*(1, 132) = 0.006, *p* = 0.939, η_p_^2^ = 0.000, indicating that dispositional risk orientation did not directly influence post-decision satisfaction. Moreover, the interaction between anticipated regret and risk preference was not significant, *F*(1, 132) = 0.201, *p* = 0.655, η_p_^2^ = 0.002. This finding suggests that the positive effect of anticipated regret on satisfaction was consistent across individuals with different levels of risk preference (Figure 5).

Overall, the results support the view that anticipated regret plays a constructive role in shaping subjective evaluations of decision outcomes, independent of individual differences in risk-taking tendencies. This has potential implications for decision-making interventions, where prompting individuals to mentally simulate possible regret may serve as an effective strategy for improving post-choice satisfaction, regardless of personality traits.

### 3.5 Summary

Participants in the anticipated regret condition demonstrated greater risk aversion and reported higher levels of choice satisfaction compared to those in the no-regret condition, reinforcing the regulatory function of anticipated regret in decision-making. These findings are consistent with the results observed in Study 1. While individual differences in risk preference significantly influenced decision behavior, no interaction was found between risk preference and anticipated regret. This indicates that the effect of anticipated regret on decision-making is relatively stable across individuals with different levels of risk tolerance.

It is worth noting, however, that Study 2 focused primarily on risk preference as a dispositional factor, which may not fully capture the impact of external situational variables on decision-making strategies. To address this limitation, Study 3 introduces time pressure as an external manipulation to examine whether the influence of anticipated regret on risk behavior and choice satisfaction persists when cognitive resources are constrained. This approach aims to provide a more comprehensive understanding of how affective forecasting operates as a regulatory mechanism under varying environmental conditions.

## 4 Study 3: Materials and methods

### 4.1 Participants

The general methodology was identical to Study 1, except for the following differences. *A priori* power analysis using G*Power 3.1.9.7 indicated that a minimum of 128 participants was needed to detect a medium effect size (*f* = 0.25) with *α* = 0.05 and power = 0.80. A total of 165 adult participants completed the study. Four participants were excluded due to incomplete data or abnormal responses, yielding a final sample of 161 (84 females; *M* = 21.59 years, *SD* = 4.59). Participants were randomly assigned to one of four experimental conditions: no time pressure with no anticipated regret (*n* = 41), no time pressure with anticipated regret (*n* = 42), time pressure with no anticipated regret (*n* = 38), and time pressure with anticipated regret (*n* = 40).

### 4.2 Design

This study employed a 2 (anticipated regret: present vs. absent) × 2 (time pressure: present vs. absent) between-subjects factorial design. The main difference from Study 1 was the addition of a time pressure manipulation, enabling the investigation of whether time pressure affects the extent to which anticipated regret influences decision-making behavior and post-decision evaluations.

### 4.3 Materials and procedure

The main difference from Study 1 was the introduction of a time pressure manipulation. Participants in the time pressure condition were required to make each inflation decision within 3 s, with failure to respond in time resulting in automatic termination of the current trial. Participants in the no time pressure condition completed the task without any time constraints. Otherwise, the experimental paradigm, emotional induction, BART procedure, and attention checks were identical to Study 1 (Suri and Gross, 2012).

### 4.4 Results and analysis

Data were analyzed using two-way analyses of variance (ANOVAs) to examine the main and interaction effects of anticipated regret (present vs. absent) and time pressure (present vs. absent) on the same outcome variables.

#### 4.4.1 Risk aversion analysis

A two-way ANOVA revealed a significant main effect of anticipated regret, *F*(1, 157) = 12.06, *p* < 0.001, η_p_^2^ = 0.071. Participants exposed to the anticipated regret condition (time pressure: *M* = 5.45, SD = 3.08; no time pressure: *M* = 5.57, SD = 1.99) exhibited significantly fewer balloon pumps on unexploded balloons compared to those in the no-regret condition (time pressure: *M* = 5.74, SD = 2.26; no time pressure: *M* = 7.90, SD = 2.12), indicating that anticipated regret reliably increased risk-averse behavior. These results are consistent with those of Studies 1 and 2, reinforcing the role of anticipated regret in promoting risk-averse behavior. A significant main effect of time pressure also emerged, *F*(1, 157) = 9.08, *p* = 0.003, η_p_^2^ = 0.055. Participants under time pressure showed lower risk-taking than those in the no time pressure condition, suggesting that time pressure fosters more cautious decision strategies.

**Figure 6:**
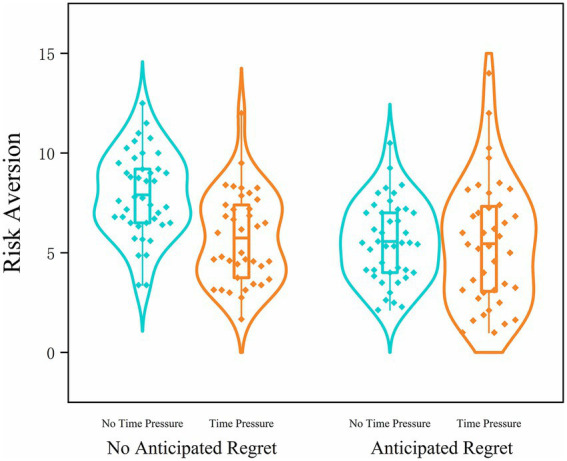
Violin plots of risk-averse behavior in Study 3 under different anticipated regret (present vs. absent) and time pressure (present vs. absent) conditions.

Crucially, the interaction between anticipated regret and time pressure was significant, *F*(1, 157) = 7.37, *p* = 0.007, η_p_^2^ = 0.045. Simple effects analyses, conducted with Bonferroni correction, examined the interaction in both directions. First, across time pressure levels, anticipated regret significantly reduced balloon pumping under no time pressure, *F*(1, 157) = 19.76, *p* < 0.001, but this effect was not significant under time pressure, *F*(1, 157) = 0.28, *p* = 0.60. Second, across anticipated regret conditions, time pressure significantly reduced balloon pumping in the no-regret condition, *F*(1, 157) = 16.09, *p* < 0.001, but had no significant effect in the anticipated regret condition, *F*(1, 157) = 0.05, *p* = 0.83. These findings indicate that the regulatory influence of anticipated regret on risk-taking diminishes under time-constrained conditions, whereas time pressure primarily affects risk-taking when anticipatory regret is absent. One plausible explanation is that time pressure limits individuals’ capacity for prospective emotional simulation, thereby reducing the influence of anticipatory emotions in guiding decision-making (Figure 6).

#### 4.4.2 Choice satisfaction analysis

A significant main effect of anticipated regret was found, *F*(1, 157) = 4.44, *p* = 0.037, η_p_^2^ = 0.028. Participants in the anticipated regret condition (time pressure: *M* = 4.60, SD = 1.39; no time pressure: *M* = 4.64, SD = 1.32) reported significantly higher levels of choice satisfaction than those in the no-regret condition (time pressure: *M* = 4.50, SD = 1.43; no time pressure: *M* = 3.83, SD = 1.36). This result supports the notion that anticipating regret prior to decision-making can positively influence individuals’ subjective evaluation of their choices, in line with the findings from Studies 1 and 2.

**Figure 7:**
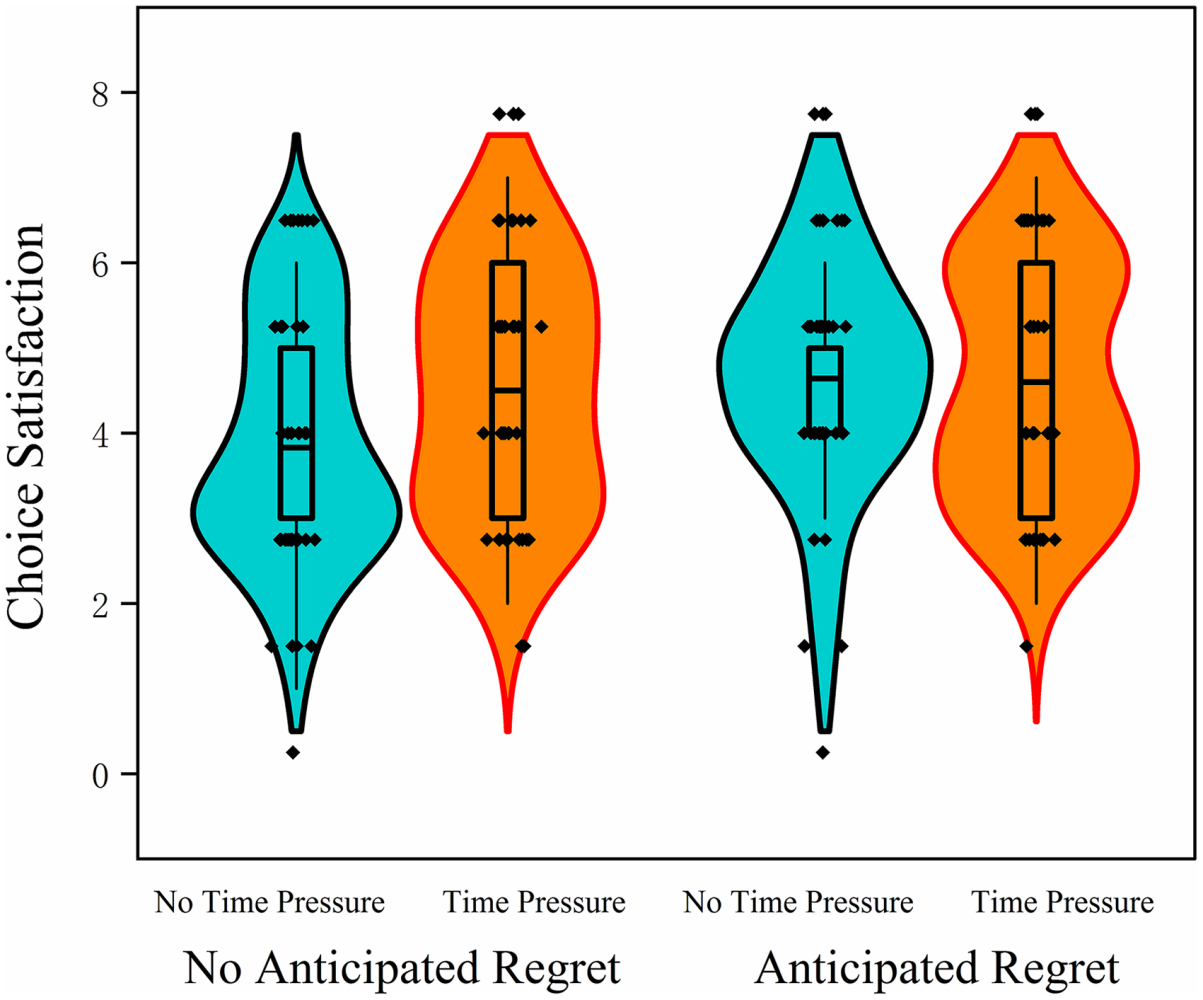
Violin plots of choice satisfaction in Study 3 under different anticipated regret (present vs. absent) and time pressure (present vs. absent) conditions.

In contrast, the main effect of time pressure was not significant, *F*(1, 157) = 2.10, *p* = 0.15, η_p_^2^ = 0.013, indicating that time constraints did not exert a direct effect on participants’ post-decision satisfaction. The interaction between anticipated regret and time pressure was also not significant, *F*(1, 157) = 2.71, *p* = 0.102, η_p_^2^ = 0.017, suggesting that the effect of anticipated regret on satisfaction remained stable regardless of the presence or absence of time pressure. These findings indicate that, while anticipated regret consistently enhances satisfaction with one’s decisions, time pressure does not appear to significantly alter this relationship (Figure 7).

### 4.5 Summary

The findings of Study 3 demonstrate that anticipated regret significantly increased risk-averse behavior, but this effect was primarily evident in the absence of time pressure. When participants were placed under time constraints, the regulatory influence of anticipated regret on risk-taking behavior was markedly diminished. This pattern suggests that time pressure may hinder individuals’ ability to engage in forward-looking emotional simulation, thereby impairing the activation of anticipated regret during the decision-making process. These results underscore the boundary conditions under which anticipated emotions operate and highlight the importance of cognitive resources in enabling their influence on decision strategies.

## 5 Discussion

This study systematically examined the influence of anticipated regret on risk-averse behavior and choice satisfaction across three experiments, while also exploring the moderating effects of individual risk preference and time pressure. The results consistently support the notion that anticipated regret serves a regulatory function in decision-making. Furthermore, the findings reveal both the stability of this emotional influence across different levels of risk preference and its susceptibility to situational factors such as time constraints. These outcomes contribute valuable empirical evidence to the understanding of how anticipatory emotions shape decision processes, and they suggest practical implications for developing interventions aimed at moderating risk-taking behaviors and improving satisfaction with decisions.

### 5.1 The influence of anticipated regret on risk-averse behavior

Across the three studies, anticipated regret consistently promoted risk-averse behavior in decision-making tasks. In Study 1, participants primed with anticipated regret made significantly fewer balloon pumps in the BART task compared to those in the control group, indicating that the mere activation of regret anticipation can lead to more cautious choices. Study 2 extended this finding by including individual risk preference as a trait variable. Results showed that while risk preference had a significant main effect on risk-taking, the effect of anticipated regret was especially pronounced among individuals with lower risk preference, suggesting that trait-level factors can amplify the behavioral impact of emotional anticipations. Study 3 introduced time pressure as a situational factor and demonstrated that the risk-reducing effect of anticipated regret was evident only when decision-makers were not under time constraints. Under time pressure, this regulatory effect diminished and lost statistical significance. Together, these results support prior theoretical frameworks that emphasize the regulatory function of forward-looking emotions (Zeelenberg and Pieters, 2007) and their interaction with both dispositional and contextual variables (Lerner et al., 2015; Suri and Gross, 2012).

Taken together, the findings suggest that although anticipated regret is a negatively valenced emotion, it serves a constructive role in encouraging more conservative decision strategies. However, its effectiveness appears primarily contingent upon contextual affordances (i.e., time pressure), while the role of individual differences requires further investigation. The consistent pattern across studies underscores the robustness of this emotional influence, while also pointing to its complexity. Specifically, the interaction between emotional anticipation, personality traits, and situational demands illustrates the multifaceted nature of decision-making under risk. These insights contribute to a growing body of literature on emotion–cognition interactions and highlight the importance of integrating emotional, dispositional, and contextual perspectives in models of decision-making.

This integration is novel in that it demonstrates the boundary conditions of anticipated regret more clearly than previous studies. Whereas earlier work often emphasized its universal risk-reducing effect, our findings highlight that such effects may weaken or even disappear when decision-makers face time pressure. This contextual contingency underscores the importance of moving beyond main effects to a more nuanced understanding of anticipatory emotions in action.

### 5.2 The influence of anticipated regret on choice satisfaction

Across all three studies, anticipated regret consistently exerted a positive influence on participants’ post-decision satisfaction. Participants in the anticipated regret condition reported higher levels of choice satisfaction compared to those in the no-regret condition, regardless of individual differences in risk preference (Study 2) or the presence of time pressure (Study 3). These findings suggest that although regret is generally perceived as a negative emotion, its anticipatory form may serve a constructive role in the decision-making process. Specifically, the activation of anticipated regret may prompt individuals to make choices more aligned with their personal goals and values, thereby enhancing their subjective appraisal of decision outcomes (Connolly and Zeelenberg, 2002).

Importantly, the results of Study 3 demonstrate that this positive effect on satisfaction remained robust even under time-constrained conditions. One possible explanation is that satisfaction with a decision is shaped not only by the objective consequences but also by psychological factors such as perceived control, decision rationality, and the congruence between expectations and choices (Botti and McGill, 2006). Even when cognitive resources are limited, the pre-decisional engagement with anticipated emotions may continue to support a sense of coherence and internal justification, ultimately leading to more favorable post-choice evaluations.

By showing that anticipated regret enhances satisfaction even under time pressure, this research extends prior work that primarily documented its role in unconstrained settings. This suggests that anticipated regret may serve a broader adaptive function—supporting psychological coherence and post-choice justification—even when individuals have limited time to deliberate. This novel insight bridges the literature on decision satisfaction with research on bounded rationality under time constraints.

### 5.3 Limitations and future research directions

While this study provides a systematic examination of the influence of anticipated regret on risk-related decision-making, several limitations warrant consideration. First, although the Balloon Analog Risk Task (BART) offers strong experimental control and moderate ecological validity, it remains a simplified, laboratory-based paradigm that may not fully reflect the complexity of real-world decision-making—particularly in scenarios involving multiple decision stages, interpersonal dynamics, or emotionally charged outcomes. Second, the study focused primarily on behavioral indicators and subjective reports, without directly assessing the underlying cognitive and neural mechanisms. Previous research has demonstrated that regions such as the prefrontal cortex and anterior cingulate cortex are involved in processing anticipated regret (Coricelli et al., 2005), indicating that this emotion engages a network of cognitive-emotional integration processes that remain unexplored in the current work. Third, the sample consisted predominantly of young adults with relatively homogeneous demographic characteristics, limiting the generalizability of the findings. Potential influences of gender, cultural background, and cognitive style on the experience and impact of anticipated regret were not systematically examined. Prior cross-cultural studies have suggested that cultural orientation significantly shapes the contexts in which regret is experienced and the intensity of the emotional response. For example, Komiya et al. (2011) and Hur et al. (2009) found that individuals from East Asian cultures, such as Korea, are more likely to experience regret in interpersonal contexts, whereas individuals from Western cultures, such as the United States, tend to report regret more frequently in autonomous, self-related decisions. These cultural differences highlight the need to examine how anticipated regret operates within broader sociocultural frameworks.

Future research should consider several directions to extend these findings. First, employing more ecologically valid decision paradigms—such as multiplayer economic games or long-term investment simulations—would help capture the social and dynamic dimensions of regret-based decision-making. Second, the integration of neuroscientific methods, including event-related potentials (ERP) and functional magnetic resonance imaging (fMRI), could elucidate the neural mechanisms underlying anticipated regret and its interaction with attentional control and executive functioning systems. Third, future studies should incorporate a wider array of individual difference variables, such as personality traits, cultural identity, and emotion regulation strategies, to construct a more comprehensive emotion–cognition–behavior model. Finally, longitudinal designs may be particularly valuable in examining how anticipated regret influences the formation of decision-making habits and adaptive strategies over time.

### 5.4 Theoretical significance and contributions

The present findings can be meaningfully interpreted within core theoretical frameworks of risky decision-making, particularly regret theory (Zeelenberg and Pieters, 2007) and decision affect theory (Mellers, 2000). These frameworks emphasize that anticipatory emotions not only guide behavioral choices but also shape post-decision evaluations.

First, the current study provides empirical evidence that anticipated regret significantly increases risk-averse behavior and enhances choice satisfaction. This directly supports the central claim of regret theory—that the anticipation of negative affect regulates decision tendencies to avoid future regret—and also corroborates decision affect theory by showing that anticipatory emotions influence both behavioral choices and subsequent subjective experience. These findings highlight that emotions in decision-making are not merely reactive but serve as proactive regulatory mechanisms, optimizing behavior by forecasting potential outcomes. Second, the study demonstrates the adaptive value of anticipated regret. Evidence across contexts indicates that anticipated regret consistently suppresses high-risk behavior while enhancing post-decision satisfaction. This suggests that anticipatory emotions exhibit both stability and functionality in risky decisions, guiding individuals to avoid potential losses and simultaneously promoting more rational and beneficial decision experiences. This insight extends existing theoretical models by emphasizing the dual role of anticipatory emotions in both behavioral regulation and affective evaluation. Finally, by linking behavioral choices with post-decision satisfaction, the study provides a more integrative perspective on emotion–cognition interactions. The results show that anticipatory emotions shape immediate decision behavior while also influencing subsequent subjective evaluation, indicating that risk decision models should incorporate both behavioral guidance and emotional feedback. This integrative approach deepens our understanding of the mechanisms of anticipated regret and offers a theoretical foundation for future research on the functions of emotions in complex decision-making contexts.

## 6 Conclusion

This study demonstrated that anticipated regret significantly promotes risk-averse behavior and enhances choice satisfaction. Across all three experiments, participants experiencing anticipated regret made more conservative decisions and reported greater satisfaction with their choices. While individual risk preference influenced risk-taking behavior, it did not moderate the effect of anticipated regret. In contrast, time pressure both reduced risk-taking and weakened the impact of anticipated regret, suggesting that sufficient cognitive resources are essential for anticipatory emotions to exert their regulatory function. These findings highlight the stable yet context-sensitive role of anticipated regret in decision-making.

## Data Availability

The datasets presented in this study can be found in online repositories. The names of the repository/repositories and accession number(s) can be found at: https://osf.io/ja547/.

## Appendix

### Risk Propensity Scale

Please indicate the extent to which you agree or disagree with the following statement by putting a circle around the option you prefer. Please do not think too long before answering; usually your first inclination is also the best one.

**Table 1:**

1. Safety first.								
Totally disagree	1	2	3	4	5	6	7	Totally agree
2. I do not take risks with my health.								
Totally disagree	1	2	3	4	5	6	7	Totally agree
3. I prefer to avoid risks.								
Totally disagree	1	2	3	4	5	6	7	Totally agree
4. I take risks regularly.								
Totally disagree	1	2	3	4	5	6	7	Totally agree
5. I really dislike not knowing what is going to happen.								
Totally disagree	1	2	3	4	5	6	7	Totally agree
6. I usually view risks as a challenge.								
Totally disagree	1	2	3	4	5	6	7	Totally agree
7. I view myself as a…								
Risk avoider	1	2	3	4	5	6	7	Risk seeker
